# Program Evaluation of Environmental and Policy Approaches to Physical Activity Promotion in a Lower Income Latinx School District in Southeast Los Angeles

**DOI:** 10.3390/ijerph17228405

**Published:** 2020-11-13

**Authors:** Anne L. Escaron, Corina Martinez, Monica Lara, Celia Vega-Herrera, Denise Rios, Marielena Lara, Michael Hochman

**Affiliations:** 1 AltaMed Institute for Health Equity, AltaMed Health Services Corporation, Los Angeles, CA 90040, USA; cvherrera@altamed.org (C.V.-H.); DeRios@altamed.org (D.R.); 2 Health Education and Wellness Department, AltaMed Health Services Corporation, Los Angeles, CA 90040, USA; cormartinez@altamed.org (C.M.); mlara1@students.llu.edu (M.L.); 3 RAND Corporation, Santa Monica, CA 90407-2138, USA; lara@rand.org; 4 University of Southern California (USC) Gehr Family Center for Health Systems Science & Innovation, Keck Medicine of USC, Los Angeles, CA 90033, USA; Michael.Hochman@med.usc.edu

**Keywords:** students, boys, girls, leisure-time physical activity, wellness policy, moderate to vigorous physical activity (MVPA), policy, systems, environmental strategies

## Abstract

There is alarming population wide prevalence of low adolescent physical activity as this represents a risk factor for later chronic disease development. There is evidence to suggest that schools with strong wellness policies have students that are more frequently active. We designed an intervention to enhance students’ physical activity levels in five majority Latinx, underserved school districts. Evaluation consisted of assessment of written quality of school-district wellness policies; observation of student’s physical activity during leisure times; and after-school program practices and policies. We examined one of these district’s results more closely, the only participating district with a community coalition, and extracted lessons learned. On the physical activity section of the wellness policy, this district covered a moderate extent of recommended content areas using weak language. Compared to previous reports, we identified low vigorous activity levels for girls and boys at baseline (respectively, 12% and 18%). Finally, we identified that of four after school program sites assessed at baseline, no program reported the recommended 50% or more of program time dedicated to physical activity. Based on these evaluation findings, additional strategies are urgently needed to encourage all students and particularly more girls to be physically active throughout the school day.

## 1. Introduction

Children and adolescents ages 6 through 17 years should do 60 min (1 h) or more of moderate-to-vigorous physical activity daily [1]. This activity should include aerobic activity as well as age-appropriate muscle- (climbing or doing push-ups) and bone-strengthening (running or jumping) activities. Despite Healthy People 2020 setting objectives, only 20% of U.S. adolescents report sufficient activity to meet the relevant aerobic and muscle-strengthening guidelines [1,2,3]. This population wide inactivity represents a risk factor for chronic disease development later in life and has been addressed through national physical activity guidelines and recommendations by the Surgeon General to foster environmental change to prevent obesity [1,4]. Whereas type 2 diabetes was previously observed primarily among adults, it has become more common among children and adolescents [5,6,7]. In 2001, the prevalence of type 2 diabetes in a sample of U.S. persons aged 10–19 years was 0.42 cases per 1000 persons and was greatest among Asian/Pacific Islander, black, Hispanic, and American Indian persons [8,9]. Moreover, there is evidence to suggest that a greater percentage of adolescents from families in poverty are obese (23%) compared with those from families not in poverty (14%). {Poverty was defined as the poverty-income ratio, which is the ratio of a family’s income to the U.S. Census Bureau’s poverty threshold. The threshold varies with the number and ages of family members and is revised yearly} [10]. Consistent with an ecologic approach for addressing these health disparities, elementary and secondary (Kindergarten-12th grade; K-12) schools represent a promising setting in which to increase physical activity levels because students spend on average six hours there daily most days of the week during the academic year [11,12,13,14,15,16,17,18,19]. Leading the charge and among the most comprehensive of these studies, the Child and Adolescent Trial for Cardiovascular Health (CATCH) study, adopted a social psychology framework for behavior change representing a departure from health education based approaches [20]. Based on this and other studies, the Community Preventive Services Task Force (CPSTF) rates healthy eating interventions combined with physical activity interventions in schools with sufficient evidence to increase physical activity levels [1,5,17]. While a robust literature supports the impact of these studies, further research is urgently needed to disseminate these findings in communities disproportionately impacted by pediatric obesity. Supervision, equipment, and structured programs are among some of the school factors reported to most influence students’ physical activity levels, with evidence additionally suggesting that schools addressing these factors through strong and comprehensive wellness policy language tend to have healthier students [21,22].

In the United States, the Child Nutrition and Women, Infants and Children (WIC) Reauthorization Act of 2004 required school districts participating in the United States Department of Agriculture (USDA)’s school meal program to create a wellness policy and to provide a framework for wellness promotion strategies [23]. These policies provide school districts with the opportunity to incorporate CPSTF recommendations such as enhanced school-based physical education (PE) and active travel to school providing some accountability for eventual implementation of these approaches [24,25,26]. Strong and comprehensive wellness policies set nutrition education and physical activity goals for the district, and they have the potential to shape the school environment to support all students to be moderately-to-vigorously active [27,28,29]. Practically speaking, these wellness policies support best practices, such as wellness councils, which give school communities a forum for teachers, school staff, students, and parents to collectively write the wellness policy and articulate the targets and priorities that support more opportunities for youth to be physically active throughout the school day [30,31,32]. Moreover, the health culture built by such policies, represent critical determinants to driving increased student vigorous physical activity [33,34,35,36]. In districts with > 50% of students eligible for Free or Reduced-Price Lunches (termed high FRPL), overall wellness policy quality was positively associated with both student mean Body Mass Index percentile and mean percent overweight or obese. Wellness policy Physical Education and Physical Activity subscale scores were also positively associated with the mean days per week students engaged in physical activity for ≥ 60 min in high FRPL districts and in low FRPL districts (<35% eligible) [22]. Policy level initiatives represent a critical strategy and may include PE-related laws, which have broad reach and impact frequency and duration of PE [37,38].

As noted above, living in poverty may predispose adolescents for obesity. There is evidence to suggest that school based interventions in under resourced school districts can contribute to positive health outcomes by increasing physical activity levels. El Paso CATCH intervention schools slowed the epidemic increase in risk of overweight or overweight seen in control school children [11]. Moreover, and of particular relevance to this study, schools in lower-income neighborhoods were reported less likely to have physical education teachers and fewer physical activity supportive practices than did higher-income schools [39]. High-socioeconomic status (SES) schools were much more likely to have a Physical Education (PE) teacher than were low-SES schools. While low-SES schools without a PE teacher were still providing some, albeit fewer, opportunities for physical activity this suggests that a majority of the obligation of providing PE and physical activity opportunities was fulfilled by classroom teachers. Children at low-SES schools had five fewer minutes per day of moderate to vigorous physical activity (MVPA) than did those at high-SES schools, although this finding was non-significant (likely due to lack of power). {The MVPA measure was created by computing the proportion of students in the walking and very active categories and represents an important metric that researchers report in order to compare school-wide physical activity levels} [21]. These findings highlight the critical role that PE teachers play in children’s physical activity and the need for national policies and funding to support hiring PE teachers, particularly in low-SES schools [39]. While schools may often provide additional activity spaces for their neighboring community, majority non White and lower-income schools were less likely to have shared use agreements (a process where the school opens its facilities/properties for recreational use outside of school hours) [40].

In 2014 a federally qualified health center (FQHC) was awarded federal funding to implement and evaluate policy and environmental strategies, consistent with a multilevel ecological framework, to increase students’ physical activity levels in Los Angeles County [41]. This county is divided into eight Service Planning Areas (SPA), geographic areas, primarily for the planning and delivery of health and social services [42]. As the FQHC serves a majority of patients in Southeast Los Angeles, these grant funded efforts were focused on cities within Service Planning Area (SPA) 7 with a total population of 1,322,943 and 12 public school districts; where 31.2% of children ages 6-17 years obtained recommended amount of aerobic exercise weekly (≥60 min daily) [43,44,45,46]. Only 19.9% of children ages 6–17 years obtained recommended amount of aerobic and muscle-strengthening each week; below the Healthy People 2030 target goal of 24.1% [3,47]. The purpose of this study was to report on a subset of a larger healthy eating, active living community health initiative within SPA7 which included 19 elementary and secondary schools across five school districts and was previously reported [48,49]. The purpose of this case study was to report on one of these district’s efforts at enhancing students’ physical activity levels by presenting data from physical activity assessments at three levels ((1) district-level assessment of the written quality of the wellness policy; (2) school level observation of student’s leisure time physical activity levels during the school day; (3) after-school program site practices and policies), and extrapolate lessons learned and recommendations for future research [30]. In short, we present pre/post intervention results of school contextual factors and students’ leisure time physical activity levels.

## 2. Materials and Methods

School participation criteria included: (1) located in SPA 7 majority Latinx and low-income, (2) willingness to designate a school-level contact to coordinate efforts, and (3) agree to start a school wellness committee or nominate an existing school committee [50]. Through our healthy eating active living initiative with 19 schools, we reached 43.5% of the majority Latinx and low-income population in a subset of predefined zip codes. The district reported on in this study is composed of four schools (Kindergarten-8th grade; K-8) with a student population of 1544 students that was 96% Latinx, 47% female, and 86% eligible for FRPL.

### 2.1. Design of the School Intervention

From August 2015 through June 2018, FQHC staff collaborated with an already established district-wide school wellness committee (SWC), which was composed of principals, school staff, parents, and representatives from professional associations, regional foundations/corporations, and local/county government. A coordinator, funded by a regional foundation, and the district’s superintendent led this community coalition. FQHC staff completed the Alliance for a Healthier Generation’s Healthy Schools Program Assessment for each school, which identified criteria for a healthy school environment and served as a guide for policy and practice changes at the school-level [51]. Through this assessment, the SWC engaged in an action planning process to identify one or two wellness promotion goals to achieve within one academic school year. Wellness goals typically included one of the following: (1) meet state-standards for minimum required physical education minutes; (2) update the district’s wellness policy to reflect state and federal wellness policy requirements; (3) update the district’s wellness policy to reflect activities presently implemented by all schools; (4) promote healthy foods and beverages during and after the school day. Over the course of the intervention period, FQHC staff provided the following at the school level: refresher trainings for teachers on the physical activity curriculum used by the school district; one-page wellness policy flyers for communicating with parents; healthy eating and physical activity signage. At the district level, a SWC sub-committee was created to guide a wellness policy revision session which took place January 2017 and was previously reported [30]. Briefly, the SWC sub-committee discussed 47 of 54 wellness policy items (including 11 of 15 items from the physical education section) that could be improved and developed new language for 39 of 47 items. The SWC sub-committee presented these revisions to the school district’s board, but they were never formally approved during the grant period due to additional budget items that leadership wanted to address simultaneously.

### 2.2. Instruments

The three major components of the evaluation were (1) a district-level comprehensive assessment of the written quality of the wellness policy (WellSAT 2.0); (2) school level observation of student’s physical activity levels using the System for Observing Play and Leisure Activity in Youth, SOPLAY, instrument; (3) after-school program school site level assessment of program practices and policies using the Healthy Afterschool Activity and Nutrition Documentation, HAAND, instrument [28,52]. We previously reported the methodology of the nutrition sections of the wellness policy assessment and after school program practices and policies evaluation and provide brief summaries below for the relevant physical activity components of these instruments [30,49].

Briefly, we obtained the district’s wellness policy on-line and completed a baseline assessment July 2015 (Table 1) [53]. As the wellness policy was not formally approved through a School Board vote, no follow up assessment was completed. WellSAT 2.0 includes one physical activity section: Physical Education and Physical Activity (PEPA, 20 items; Supplementary Table S1) [53]. Items included alignment of written physical education curriculum with standards, time per week of physical education instruction, and active transport among others. Since the “time per week of physical education instruction” item was only relevant for scoring within high schools and as no high schools were included in the sample, this item was not scored and results are reported for 19 items. According to WellSAT 2.0 scoring rubric, each item was scored 0 (Not Mentioned); 1 (Weak Statement); or 2 (Meets/Exceeds Expectations). Two trained policy analysts rated the wellness policy independently and assigned each WellSAT item a score of 0, 1, or 2. After analyzing the policy, both analysts met to review scores for all items. Discrepancies in scores were conferred with a third analyst and a final score was decided.

Consistent with the SOPLAY protocol, trained data collectors made systematic and periodic scans of individuals and contextual factors within predetermined target areas including marked courts, grassy fields, and blacktop [48]. During a scan, the activity of each student is coded as Sedentary, Walking, or Very Active using a three-way counter—a first scan is completed to gain a count for female students in each of these activity categories while a second scan is completed to gain a count of male students in each of these categories. Contextual factors or school area features included accessibility, not locked; usability, not excessively wet or windy; supervision, school personnel available to students in case of emergency; organized-school personnel leading, instructing, or organizing students in physical activity; availability of equipment (i.e., balls, jump ropes, or other loose equipment to promote physical activity). The prominent activity students were engaged in was also recorded (i.e., basketball, volleyball, and dance) and these are previously reported. To ensure consistency in timing across schools, scans were completed before school, during school (typically lunch recess), and after school over at least three randomly selected days within a two week period per school. For all four schools, we completed the baseline assessment September–October 2015; follow-up 1 in April–May 2016; follow-up 2 in May 2017.

We assessed the extent to which after school programs implemented physical activity policies and practices using the HAAND instrument [52]. In order to obtain school site level after-school program information, we asked the after-school regional manager to designate a site supervisor for each of the four schools who had working knowledge of the program and led or facilitated the program. Based on the regional manager’s recommendation, we invited one after-school program supervisor per school to complete the brief interview on campus (15–30 min), and we then completed a document review to supplement responses obtained during this interview. HAAND captures after school program’s physical activity practices and policies across seven domains and 11 items. Here we present a subset of the three items drawn from the schedule of physical activity domain relevant to physical activity promotion: time allocated (no scheduled time; less than 25% of schedule; 25–49% of schedule; or 50% or more of schedule, respectively, assigned a score of 0, 1, 2, or 3 points according to the HAAND rubric); types of activities (free play; limited # of activities one to two structured activities; or diverse range of structured activities ≥3, respectively, assigned a score of 0, 1, or 2 points); and equity (activities favor single gender or activities appeal to both genders, respectively, assigned score of 0 or 1 point; Supplementary Table S1). Per the HAAND protocol, we also report on one observation per school site during after-school program time of whether students were observed being physically active in an organized activity led by program staff (yes, if students observed with staff engaged in an activity; no, if zero students were observed in designated activity area) and what types of physical activities (free play or staff-led games) were observed. We completed evaluation activities prior to intervention implementation (i.e., baseline assessment September 2015) and completed follow-up interviews April 2016.

These activities were reviewed (#1-887808-1) and deemed exempt by Western Institutional Review Board. In addition, the participating school district reviewed and approved these activities prior to principal outreach.

### 2.3. Data Analysis

For WellSAT2.0, comprehensiveness is calculated by counting the number of items in each section rated as “1” or “2,” dividing this number by the number of policy items in the section, and multiplying this number by 100. The comprehensiveness score reflects the extent to which recommended content areas are covered in the policy. Strength is calculated by counting the number of items in each section rated as “2,” dividing this number by the number of policy items in the section, and multiplying this number by 100. The strength score captures the degree to which policies included specific and firm language. Both scores range from 0 to 100, with lower scores indicating less content and weaker language (e.g., using “will” statements, rather than “may” statements), and higher scores indicating more content and use of specific and directive language.

In tabulating SOPLAY observations, the proportion of students that were sedentary, walking, or vigorous was computed by tallying counts of girls or boys by activity level to obtain a summary score. This summary score was summed to obtain a total count of girl or boys. By activity level, the respective summary score was divided by the total in order to obtain a proportion of total students by activity level. A higher percentage indicates a greater proportion of students in that activity level. As our program was designed to promote leisure time physical activity, we were looking for increases in walking and vigorous activity with decreases in sedentary activity from baseline to follow-up.

For HAAND, we report school level average score by item relative to points possible (time allocated = 3 points; types of activities = 2 points; and equity = 1 point). A higher score indicates a program that is more aligned with national physical activity guidelines and best practices. All calculations were performed in Microsoft Excel 2010 (Microsoft Corporation, Redmond, WA, USA).

## 3. Results

### 3.1. WellSAT 2.0

A comprehensiveness score of 63% and a strength score of 21% (i.e., how firmly the content was stated) was calculated for the PEPA section of WellSAT2.0 (Table 2). Results were shared with school representatives (e.g., school district Superintendent, teachers, parents) during SWC meetings to highlight opportunities for improvement. FQHC staff worked closely with district-level staff and the SWC-subcommittee to revise and update their district wellness policy and this process was previously reported [30].

### 3.2. SOPLAY

School areas designated for students’ leisure time physical activity (Table 3) were accessible and usable. At baseline (8% before school, 54% lunch recess, 38% after school), areas were 62% supervised and 24% equipped, however, very low organized activity was observed. Given the methods and small sample, changes in physical activity cannot be made with confidence—nonetheless girls (12%) were less vigorously active than boys (18%) at baseline (Table 4), whereas at follow-up 1 girls (17%) and boys (22%) increased their vigorous activity levels. Girls’ walking increased from baseline (46%) to follow up 2 (54%) with a similar pattern observed for boys’ walking.

### 3.3. HAAND

At baseline, we conducted interviews with each of the four schools’ after school staff. At follow-up, interviews with two of the schools’ after school staff were completed (the other two did not respond to requests for the follow-up interview). At baseline and follow up (Table 5), programs delivered activities that appeal to females and males offering a diverse range of activities. At both time points, programs fell short of recommendations with no more than 25–49% of schedule time allocated to physical activity. When the after school programs were observed for students’ physical activity, 1 of 4 programs at baseline were observed with students being active through staff-led games (monkey in the middle); at follow-up, 2 of 2 programs were observed with students active through staff-led games (basketball).

## 4. Discussion

This study presented findings from three unique assessments preceding and following an intervention to enhance students’ physical activity levels in one underserved school district. Key evaluation findings were as follows: (1) the school district’s PEPA wellness policy comprehensiveness exceeded strength, suggesting that there are opportunities to enhance the written policy language as well as prioritize incorporation of more evidence based strategies into the wellness policy; (2) during leisure times, vigorous activity levels were low for boys and girls relative to what others have reported in the literature suggesting that significantly more attention is needed to redress the lower vigorous activity levels observed in girls; (3) the after school programs offered a variety of physical activities that appeal to boys and girls, however, there is room for improvement in dedicating a greater percentage of the schedule time to physical activity [21,48,54]. Previously we reported on the FQHC team’s efforts to prioritize recommendation of evidence-based physical activity strategies into this district’s wellness policy in order to build in practices such as addressing time per week of PE instruction; recess; before/after school opportunities for physical activity. For low income districts, revising policy may be a critical approach to adopting these practices [30,55].

In the current report, we found that vigorous physical activity levels were low for boys (18%) and girls (12%) relative to what was previously found. For example, in 24 middle schools with 39% FRPL program participation, boys and girls (respectively, 29.80% and 24.60%) had higher vigorous physical activity proportions [21]. In another report, 32% of boys and 22% of girls were vigorously active—levels well above what we observed [56]. It is interesting to note that vigorous activity levels were moving in the hypothesized upward direction, which may provide evidence for the contribution a team of data collectors conducting observational assessments makes in stimulating the desired behavior. Others have formally tested the effect of an evaluation only control group [20].

A report from Australia highlights the contribution of school district resources to health behaviors, with primary school children attending high socioeconomic schools 1.71 times more likely to achieve the healthy fitness zone for cardiorespiratory fitness than primary school children attending low socioeconomic status (SES) schools. Secondary children attending high SES schools were 1.87 times more likely to achieve the healthy fitness zone for cardiorespiratory fitness, than children attending low SES secondary schools [57]. While the number of potential barriers to enhance children’s PA was higher for high compared to low SES elementary schools, low SES secondary schools reported a higher number of barriers overall than high SES counterpart schools [57]. These low SES secondary schools reported fewer barriers associated with curriculum and teaching, but more barriers associated with intrinsic student factors and school policy and environment. Of note, although it was just shy of statistical significance, in a report of 347 Nevada public elementary, middle, and high schools, schools with comparatively larger shares of Latinx students were more likely to report compromising physical education for space reasons. The authors speculated that these schools were older and originally built for smaller numbers of students, thereby placing increased demand on spaces originally intended for physical education. They go on to speculate that state and school-district policies do not require principals to schedule facilities in specific ways therefore site-based management decision-making may be driving this difference and prioritizing instruction space. Without formal subscription to physical activity practices, these considerations may go unexamined [58]. As regards school factors, we observed deficiencies in “organized” physical activity (around 1%) in contrast with one report of 13 Title I elementary schools with Latinx enrollment of at least 70% noting close to 17% of observed areas benefiting from school staff leading or officiating physical activity with students during leisure times [21].

In after school programs, we observed equity and variety in daily physical activities. However, there may be an opportunity for public health practitioners to support school and after school program leadership in offering more unstructured play activities; to train staff on how to safely use physical activity equipment, and how to manage playground behavior while also conducting engaged supervision. This is one topic districts could include in their district wellness policy that would showcase best practices and promote consistency across schools [59]. Of note, the after school program served on the district’s community coalition and likely played a part in successful outreach to staff for participating in the HAAND interviews. These partnerships contribute to synergy in school-based physical activity promotion as after school program administration is distinct from school district administration.

Further analysis is underway to determine what impact equipment had on students’ physical activity over the course of this initiative [60]. We previously reported that the availability of loose playground equipment positively impacted MVPA [48]. Previous literature has demonstrated positive outcomes among elementary aged girls when the school playground environment is enhanced during recess time and merit additional consideration. Interestingly, in previous studies rates of MVPA were higher for boys in play field areas (i.e., soccer), hard-surface play areas (i.e., basketball), and loose play equipment areas, but girls were by far the highest users of loose play equipment areas [61]. More investigation into the quantity and type of equipment is needed [21,56].

Strengths of this evaluation are its multilevel aspect describing the school environment and policy environment relying on validated instruments spanning observational methods of physical activity behaviors as well as structured interviews and document review [62]. Limitations faced while collecting after-school program data included loss of staff to follow-up interviews. Furthermore, responses during interviews could be subject to social desirability bias. While SOPLAY is widely used, the validity of observational data can be compromised by poor inter-rater reliability and biased towards activity occurring in defined activity areas. The small sample size limited our ability to present rigorous pre/post comparisons. Regardless, our initiative made important contributions characteristic of multicomponent interventions that combine school environment characteristics as well as policy elements [41,63]. Taken together, future program evaluation within low-income schools should focus on identifying modifiable social and physical school characteristics that are likely to promote physical activity and explore how these factors differ among boys and girls and by school level [64,65,66]. Researchers, advocates, and practitioners should leverage resources to advance PE related laws and policies that incentivize, systematize, and fund opportunities for all students to meet daily physical activity guidelines and reverse the trend towards inactivity [37,38]. This focus is particularly important in underserved communities given the disproportionate prevalence of pediatric obesity and chronic conditions [2,6,67].

## 5. Conclusions

Based on our work, we recommend the following to school stakeholders:

- Prioritize wellness policy revisions with diverse school stakeholders, including parents and other caregivers, in order to bolster comprehensiveness and strength of the district’s wellness policy language as well as accountability for these changes;
- Capitalize on opportunities to increase availability of school yard equipment as this strategy represents one potentially modifiable factor to enhance students’ physical activity;
- Partner with after school program decision makers to ensure that the schedule includes 50% or more of time allocated to vigorous physical activity.

## Figures and Tables

**Table 1:** Timeline of physical activity intervention and assessment components.

	Year 1				Year 2				Year 3			
Health center staff led intervention components												
School-level												
Conducted Healthy Schools Program Assessment												
Led action planning process												
Led refresher trainings with teachers on district’s physical activity curriculum												
Delivered physical activity equipment												
Posted healthy eating and physical activity signage												
District-level												
Created SWC sub-committee to inform wellness policy revision session												
Health center staff led assessments												
School-level												
SOPLAY- observation of students’ leisure time physical activity												
Baseline												
Follow Up												
HAAND- interview re after-school program practices and policies												
Baseline												
Follow Up												
District-level												
WellSAT 2.0- evaluation of written wellness policy quality												
Baseline												
Follow up ^1^												

^1^ Year 1 started August 2015. Colored space indicates quarter when activity listed took place. School Wellness Committee (SWC); System for Observing Play and Leisure Activity in Youth (SOPLAY); Healthy Afterschool Activity and Nutrition Documentation (HAAND).

**Table 2:** Physical Education and Physical Activity (PEPA) Section of WellSAT 2.0.

Rating	Item
2	There is a written physical education curriculum for grades K-12
2	The written physical education curriculum is aligned with national and/or state physical education standards
1	Addresses time per week of physical education instruction for all elementary school students
0	Addresses time per week of physical education instruction for all middle school students
N/A	Addresses time per week of physical education instruction for all high school students
0	Addresses teacher–student ratio for physical education classes
2	Addresses qualifications for physical education teachers for grades K-12
2	District provides physical education training for physical education teachers
0	Addresses physical education waiver requirements for K-12 students
1	Addresses physical education exemptions for K-12 students
0	Addresses physical education substitution requirements for K-12 students
0	District addresses the development of a comprehensive school physical activity program (CSPAP) plan at each school
1	District addresses active transport for all K-12 students
1	District addresses before and after school physical activity for all K-12 students
1	District addresses recess for elementary school students
1	Addresses physical activity breaks for all K-12 students
1	Addresses staff involvement in physical activity opportunities at all schools
0	Addresses family and community engagement in physical activity opportunities at all schools
0	District provides physical activity training for all teachers
1	Joint or shared-use agreements for physical activity participation at all schools
Comprehensiveness: 63	Strength: 21

Grades Kindergarten-12th grade (K-12).

**Table 3:** Contextual factors of observed areas at four Kindergarten-8th grade (K-8) schools at baseline and follow-up.

Features of Observed Areas	Percent %		
	Baseline	Follow-Up 1	Follow-Up 2
Accessibility	98%	96%	98%
Usable	91%	99%	99%
Supervised	62%	81%	80%
Organized	1%	1%	0%
Equipped	24%	27%	35% ^1^

^1^ Trained data collectors made scans of contextual factors including accessibility, not locked; usability, not excessively wet or windy; supervision, school personnel available to students in case of emergency; organized, school personnel leading, instructing, or coaching students in physical activity; and availability of equipment i.e., balls, jump ropes, or other loose equipment to promote physical activity. Baseline observations were completed September–October 2015; follow-up 1, April–May 2016; follow-up 2, May 2017.

**Table 4:** Physical activity levels among girls and boys during leisure times at four Kindergarten-8th grade (K-8) schools at baseline and follow-up.

Physical Activity Levels	Percent %		
	Baseline	Follow-Up 1	Follow-Up 2
School Girls			
Sedentary	42%	40%	29%
Walking	46%	43%	54%
Vigorous	12%	17%	17%
School Boys			
Sedentary	31%	30%	19%
Walking	51%	47%	56%
Vigorous	18%	22%	25% ^1^

^1^ The activity of each student was coded as Sedentary, Walking, or Very Active before school, during school (lunch recess), and after-school over at least three randomly selected days within a two week period per school. Baseline observations were completed September–October 2015; follow-up 1, April–May 2016; follow-up 2, May 2017.

**Table 5:** Healthy Afterschool Activity and Nutrition Documentation (HAAND) schedule of physical activity.

		Baseline n = 4	Follow Up n = 2
Item	Points Possible	Mean Score (SD)	Mean Core (SD)
Time allocated	3	1.3 (0.5)	2 (0)
Types of activities	2	1.8 (0.5)	2 (0)
Equity	1	1.0 (0)	1.0 (0)

## Supplementary Materials

The following are available online at https://www.mdpi.com/1660-4601/17/22/8405/s1, Table S1: Program evaluation rubric for WellSAT2.0 district wellness policy and after school program practices and policies.
