# Supplementary material for: Program Evaluation of Environmental and Policy Approaches to Physical Activity Promotion in a Lower Income Latinx School District in Southeast Los Angeles
Source: Int J Environ Res Public Health. 2020 Nov 13;17(22):8405. doi: 10.3390/ijerph17228405 (PMC7698008; doi:10.3390/ijerph17228405)
Supplement: Supplementary file 1 [file ijerph-17-08405-s001.pdf]

**Supplementary Table 1.** Program evaluation rubric for WellSAT2.0 district wellness policy and after school program practices and policies.

| Instrument | Section | Number of Physical Activity Items | Number of Physical Activity Items Scored | All Items Same Scoring | Scoring | Calculation | Interpretation |
|---|---|---|---|---|---|---|---|
| WellSAT2.0 | Physical Education and Physical Activity | 20 | 19 | Yes | 0 (Not Mentioned)<br>1 (Weak Statement)<br>2 (Meets/Exceeds Expectations) | Comprehensiveness: count the number of items rated as "1" or "2," divide this number by the number of policy items in the section, and multiply this number by 100<br>Strength: count the number of items rated as "2," divide this number by the number of policy items in the section, and multiply this number by 100 | Comprehensiveness, Strength scores range 0-100 |
| HAAND | Schedule of physical activity | 3 | 3 | No | Time allocated (0, 1, 2, or 3 points)<br>Types of activities (0, 1, or 2 points)<br>Equity (0 or 1 point) | Average of school level score | Points possible:<br>Time allocated = 3 points<br>Types of activities = 2 points<br>Equity = 1 point |

Healthy Afterschool Activity and Nutrition Documentation (HAAND).
